# The Role of Sweet Taste in Satiation and Satiety

**DOI:** 10.3390/nu6093431

**Published:** 2014-09-02

**Authors:** Yu Qing Low, Kathleen Lacy, Russell Keast

**Affiliations:** Physical Activity and Nutrition Research, School of Exercise and Nutrition Sciences, Deakin University, Burwood, Victoria 3125, Australia; E-Mails: yqlow@deakin.edu.au (Y.Q.L.); katie.lacy@deakin.edu.au (K.L.)

**Keywords:** oral sweet taste sensitivity, oral sensitivity, sensory specific satiety, satiety, obesity, BMI, sugar, sweeteners, appetite, sweet taste

## Abstract

Increased energy consumption, especially increased consumption of sweet energy-dense food, is thought to be one of the main contributors to the escalating rates in overweight individuals and obesity globally. The individual’s ability to detect or sense sweetness in the oral cavity is thought to be one of many factors influencing food acceptance, and therefore, taste may play an essential role in modulating food acceptance and/or energy intake. Emerging evidence now suggests that the sweet taste signaling mechanisms identified in the oral cavity also operate in the gastrointestinal system and may influence the development of satiety. Understanding the individual differences in detecting sweetness in both the oral and gastrointestinal system towards both caloric sugar and high intensity sweetener and the functional role of the sweet taste system may be important in understanding the reasons for excess energy intake. This review will summarize evidence of possible associations between the sweet taste mechanisms within the oral cavity, gastrointestinal tract and the brain systems towards both caloric sugar and high intensity sweetener and sweet taste function, which may influence satiation, satiety and, perhaps, predisposition to being overweight and obesity.

## 1. Introduction

Obesity represents one of the largest preventable diseases worldwide and is thought to be a key contributor to a number of major health problems, such as high blood pressure, stroke, type 2 diabetes, coronary heart disease and some forms of cancer [1]. According to the Landmark Global Burden of Disease report, obesity was emphasized as the prominent cause of disability worldwide and as a more significant health crisis worldwide than starvation and/or malnutrition [2]. Throughout the past 30 years, obesity has been a prominent problem and has increased globally in all classes of socioeconomic status in both developing and developed countries [3]. In the year 2005, the estimated total numbers of overweight and obese adults were 937 million and 396 million, respectively [4]. If current trends remain, by the year 2030, it is projected that 2.16 billion adults would be overweight and 1.12 billion adults would be obese, increasing the liability of obesity-related morbidity and mortality [4,5].

Increased energy intake, in particular greater intakes of sweet, energy-dense food, is thought to be one of the major contributors to the global rise in being overweight and obesity [5,6]. Foods that taste sweet have long been associated with dietary energy [6,7]. As an example, in the current day, excessive consumption of sugar, particularly in sugar-sweetened beverages, has been linked to the rising rates of obesity worldwide [8,9]. Refined sugars added to food and beverages have little to no nutritional value and contribute to increased energy intake [10,11]. Thus, one potential way to tackle the current obesity crisis is to reduce energy intake through high intensity sweeteners (HIS) [12,13]. Replacing sugar with HIS in a beverage or food decouples sweetness from energy value while maintaining sweetness [13,14]. Unfortunately, the scientific literature on the topic of sweetness and energy intake is divided [6]. HIS, near zero-calorie sugar replacements, were introduced before World War I [15]; however, it was not until the 1990s that HIS have been widely promoted as “healthier alternatives” in commercialized goods, such as low-calorie soft drinks, processed foods and confectionaries. Yet, the prevalence of obesity has continued to increase.

The continued increase in the prevalence of obesity worldwide despite a logical solution to decrease energy intake suggests two likelihoods. One, HIS did effectively reduce energy intake from sweet foods, and increases in obesity are due to other factors, such as the increased consumption of cheap, energy-dense foods [5]. Two, HIS did not reduce energy intake [16]. Sweetness without the associated energy may actually increase appetite and encourage consumption of other foods [17]. If the second possibility holds true, then perhaps what promotes weight gain is more complicated than decoupling taste and energy, with physiological systems demanding, via appetite, the “sweet” promise of energy being delivered. The aim of this review paper is to discuss possible associations between sweet taste function (tasting caloric sweeteners and HIS) with satiation and satiety. The possible associations between sweet taste mechanisms within the oral cavity, GI tract (GIT) and the brain systems towards both caloric sweeteners and HIS and sweet taste function will also be reviewed.

## 2. Taste and Its Function

The sense of taste is one of the traditional five senses (sight, hearing, taste, smell and touch) and refers to the sensation derived when non-volatile chemical molecules stimulate receptors sited on taste cells in the surface areas of the tongue, soft palate and the oropharyngeal region [18]. Taste is stimulated through the activation of taste receptor cells (TRC) found in the surface regions throughout the oral cavity [18]. Once the TRC are activated, electrical impulses are transmitted via the sensory afferent fibres to the brain areas involved in the cortical processing of taste, and a taste perception associated with the chemical will be experienced [19]. TRC are housed within the taste buds, which are distributed across three different types of tongue papillae (*i.e*., circumvallate, foliate and fungiform papillae) [18].

The human taste system is now widely accepted to include five taste qualities: sweet, salty, bitter, sour and umami [20]. From an evolutionary perspective, it is postulated that the human taste system functions as a gatekeeper of the digestive system to ensure that we consume essential nutrients for survival and functioning, while rejecting potentially harmful or toxic foods [20]. For example, a salty taste quality signals the presence of either sodium or minerals; umami indicates the presence of proteins; excessive sour taste signals spoiled food; bitter taste quality often indicates the presence of poisons; and sweet taste indicates the presence of carbohydrates or energy in the food [20,21]. However, from an evolutionary standpoint, it is maladaptive to recognize sweet taste from HIS, as the sweeteners do not contain energy for physiological function.

**Figure 1:**
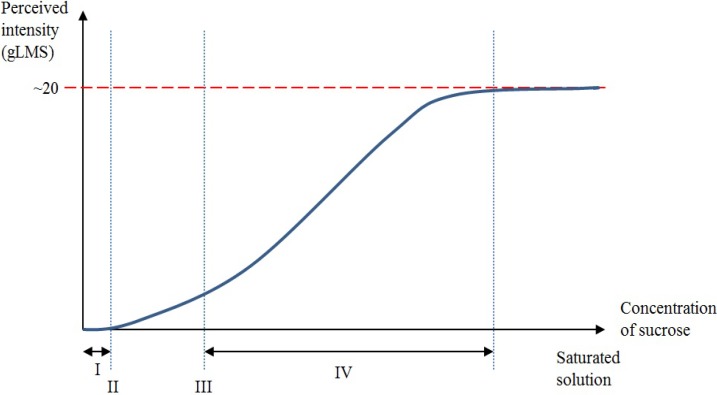
Graphic representation of the relationship between detection threshold, recognition threshold, suprathreshold intensity and chemical concentration levels. The *x*-axis of the graph represents the chemical concentration level from no concentration of sucrose in the aqueous solution (0 molar) to a saturated sucrose solution. The *y*-axis represents the general Labelled Magnitude Scale (gLMS) from no perception to a conjectural terminal threshold. (**I**) A small amount of sucrose diluted in an aqueous solution could not be detected at a low concentration. (**II**) A detection threshold is reached whereby the sucrose solution can be told apart from water. At this stage, the taste quality remains unidentified. (**III**) The recognition threshold is reached whereby the correct taste quality can be recognized. (**IV**) Suprathreshold intensity is defined as the dynamic phase where the perceived intensity of sweetness jointly increases to a hypothetical asymptote as the concentration of sucrose increases. Further increases following the dynamic phase no longer cause subsequent increases in perceived intensity.

A taste perception for a particular taste quality is experienced when the concentration of that particular solvent in the oral cavity reaches a level that activates a taste receptor [19] (Figure 1). For illustration, when only a small amount of sucrose is diluted in an aqueous solution, an individual may not be able to differentiate the aqueous solution from water [19]. As the concentration of sucrose increases, the aqueous solution can be differentiated from water, and a detection (absolute) threshold is reached [19]. However, at this stage, the individual will not be able to recognize the taste quality. As the concentration increases further, the recognition threshold will be reached when the taste quality is correctly identified (sweet in this case) [19]. As the concentration of sucrose increases even further, the perceived intensity of sweetness jointly increases to a hypothetical asymptote, where an additional increase in the concentration of sucrose no longer causes consequential increases in perceived taste intensity [19,21,22]. This dynamic phase is defined as the suprathreshold intensity perception range [19].

## 3. Peripheral and Central Mechanisms for Sweet Taste Detection

Taste processing involves a multifaceted flow of events involving taste, learning, memory and the reward systems [12]. It is thought that the individual’s ability to detect or sense sweetness in the oral cavity (initial process of sweet taste perception) is one of myriad factors influencing food acceptance, and as such, taste may play an essential role in modulating food acceptance and/or energy intake [7]. In addition to an individual’s ability to detect sweet taste within the oral cavity, emerging evidence now suggests that the sweet taste signaling mechanisms identified in the oral cavity also operate in the gastrointestinal (GI) system, which may possibly influence satiety [23]. Therefore, understanding the individual differences in detecting sweetness in both the oral and GI system towards both caloric sweeteners and HIS and the functional role of the sweet taste system may possibly be an important factor in understanding the causes for excess energy intake. In this review, evidence of possible associations between sweet taste mechanisms within the oral cavity, GIT and the brain systems towards both caloric sweeteners and HIS and sweet taste function are summarized and discussed. The possible links between sweet taste function, satiation and satiety towards both caloric sweeteners and HIS are also discussed. The term “satiation” in this review refers to the process that contributes to the cessation of a meal, whereas “satiety” is mainly associated with the post-absorptive effects of consuming foods and, therefore, will be considered as the time intermission until the next eating episode [14].

### 3.1. Peripheral Mechanisms for Sweet Taste Detection

**Figure 2:**
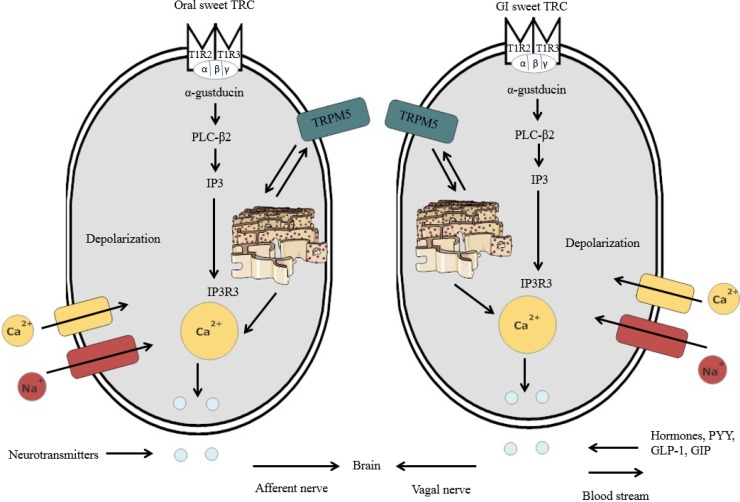
Schematic representation of lingual sweet taste receptor cells (TRC) and GI sweet TRC consisting of TIR2-T1R3 dimers. Once the sweet substances bind to the sweet chemicals, intracellular signaling elements are activated, including α-gustducin, which, in turn, activates PLC-β2. The stimulation of PLC-β2 leads to the generation of IP3, where the IP3R3 further activates the calcium ions from the endoplasmic reticulum. After the calcium ions are released, the TRPM5 channel is activated, resulting in sodium entry in the plasma membrane. Sodium entry leads to depolarization, thus inducing calcium entry through the calcium channel. The calcium ions then induce the discharge of neurotransmitters from oral sweet TRC, which are then relayed via the afferent nerve to the brain areas involved in sweet taste processing. In the GI sweet TRC, satiety hormones, such as peptide tyrosine (PYY), glucagon-like peptide 1 (GLP-1) and glucose-dependent insulinotropic peptide (GIP), are released upon secretion of calcium ions within the cell. These satiety signals are then relayed to the brain via the vagal nerve.

It has been shown that there are similarities for sweet detection in the oral cavity and GIT, between sweet oral TRC and the GI sweet TRC [24,25,26,27,28,29,30] (Figure 2). The existence of an almost identical nutrient-sensing mechanism in the oral cavity and GIT seems reasonable, given that both are part of the alimentary canal and responsible for the identification of nutrients and non-nutrients in foods [31]. Further, both the oral cavity and GIT initiate the appropriate functional responses, such as taste perception (oral cavity) and hormone release (e.g., satiety hormones) (GIT) [31].

### 3.2. Sweet Taste Detection in the Oral Cavity

The sweet TRC is a heterodimer of two G-protein coupled receptors (GPCR), T1R2 and T1R3 [32,33,34,35,36,37,38]. The T1R2-T1R3 dimer entails an extracellular region, termed the “Venus fly trap domain” (VFTD), which senses a large variety of sweet substances, such as caloric sugars (glucose, fructose and sucrose), sweet amino acids and synthetic dipeptides (e.g., aspartame) [36,38,39,40]. It is postulated that once these sweet molecules bind to the VFTD, it induces conformational changes in the VFTD, which further leads to the activation of the sweet TRC [40]. Other than the VFTD, it has been suggested that the external region of the activated sweet TRC may act as a potential activation site [41]. For instance, the C-terminal TM region of the T1R3 has been proposed to be the binding site of cyclamate, a HIS [42], whereas the cysteine-rich area of the T1R3 is also another potential contender site for the binding of sweet substances [36,41].

Once the compounds bind to the sweet TRC, intracellular signaling elements are activated, including α-gustducin, which, in turn, activates phospholipase C-β2 (PLC-β2) [43,44] (Figure 2). The stimulation of PLC-β2 leads to the generation of diacyl-glycerol and inositol-trisphosphate (IP3), where isoform 3 of the IP3 receptor (IP3R3) further mobilizes the calcium ions from the endoplasmic reticulum into the cytoplasm [45]. Once the calcium ions are released from the endoplasmic reticulum, the TRPM5 channels are activated (*i.e*., calcium-activated cation channel resulting in sodium entry) in the plasma membrane of the sweet TRC [46]. Sodium entry resulting from the activation of the TRPM5 leads to depolarization in the plasma membrane [37,47]. Consequently, this induces calcium entry through the voltage-gated calcium channel [36,46]. Once activated by sugar or HIS, sweet TRC transmit the information via sensory afferent fibres to the brain areas involved in sweet taste processing [26]. The oral sweet information is also sent to the stomach via the vagus nerve to proceed with the cephalic phase response (*i.e*., gastric juice secretion) [47]. This process further initiates functional responses in the GIT, such as glucose uptake and hormone release [26,47].

### 3.3. Sweet Taste Detection in the GIT

It is now evident that the expression of the functional sweet TRC is not restricted to the oral cavity, but is also present in other parts of the GIT, including the intestinal enteroendocrine cells [24,28] and other tissues, such as the pancreas [48]. However, individuals can only consciously perceive sweetness upon the activation of the sweet TRC in the oral cavity, because sweet TRC in the GIT and other tissues do not convey taste perception [49]. Likewise, with the oral sweet TRC, the GI sweet TRC also expresses α-gustducin, PLC-β2 and TRPM5 in the GIT (Figure 2) [50]. Upon stimulation with sugars or HIS by the GI sweet TRC, digestive and absorptive processing of the ingested food is further coordinated by the secretion of gut hormones, including PYY, GLP-1 and GIP, further regulating insulin release from pancreatic β-cells [23,50,51]. These gut hormones are associated with the metabolism of nutrients and fullness [50]. In addition to the regulation of incretins, the GI sweet TRC also controls for glucose uptake in the intestinal epithelium [52]. The glucose uptake in the GI lumen is facilitated by two types of glucose transporters: sodium-glucose transport protein-1 (SGLT-1) and glucose transporter 2 (GLUT2) [52,53,54].

### 3.4. HIS and Their Role in the GIT

As previously described, the sweet TRC can bind to sweet molecules of varying structures, including caloric sugars and a range of HIS. However, the metabolic fate of HIS varies in the GIT depending on the chemical structure of each HIS [49]. It was suggested that HIS, such as sucralose and saccharin, are not digested or metabolized in the body, whereas aspartame breaks down into smaller compounds to be digested in the intestine [55]. Given the chemical heterogeneity of these HIS, it is surprising that these sweeteners still have the ability to activate sweet TRC in the oral cavity, generating similar signals to caloric sugars, resulting in a similar sweetness signals in the brain [36,49,55]. However, the signaling transduction and downstream actions, such as satiety hormone release in the GI system, upon activation by HIS is controversial. Some studies support the hypothesis that HIS bind to the sweet TRC on the enteroendocrine cells, resulting in similar signal transduction and downstream actions to caloric sugars, such as satiety hormone secretion in humans [49,55,56]. Nevertheless, the majority of *in vivo* studies have failed to confirm this [57]. Fujita *et al.* [57] reported that despite oral taste receptor sensitivity to HIS, four different HIS (acesulfame K, stevia, sucralose and d-tryptophan) did not evoke GLP-1 or GIP release in rats. It is noteworthy that in this study, the concentrations of sweetener given were 1000-fold in excess of the concentrations used in HIS-sweetened products, such as diet soda [49]. Similarly, in human studies, HIS, such as sucralose, aspartame and acesulfame K, did not evoke any GLP-1 release [58,59,60]. The inability of HIS to initiate any GLP-1 hormones questions previous notions where sweet TRC were thought to be involved directly in GI glucose sensing [61].

Recent studies investigating the role of SGLT-1 in cultured L-cells have suggested that perhaps the SGLT-1 is more involved in sugar sensing in the GIT in comparison to the T1R2-T1R3 dimer [61]. SGLT-1 has an important function in glucose homeostasis, as it is the primary transporter of dietary sugars in the GI lumen [62] and has been found to be involved in the mechanism of glucose-induced incretin release in cultured mice L-cells [63,64]. It is important to note that Moriya *et al.* [65] did not report a marked increase in GLP-1 upon administration of HIS, such as saccharin and sucralose, to non-metabolizable SGLT-1 substrates. As SGLT-1 functions as the primary gut glucose sensor instead of sweet TRC, collectively, this further suggests that perhaps HIS are able to generate taste signals from the sweet TRC in the oral cavity, but do not stimulate gut receptor mechanisms that are involved in satiety. A full description of the signaling pathways that facilitate sweet taste perception for both caloric sugars and HIS within the GIT in human studies warrants further investigation, as it is of major importance, due to their possible involvement with GI functions, including satiety hormone release and, thus, appetite and energy intake.

### 3.5. Sweet Taste Processing in the Brain

The previous subsections have focused on the sweet taste signaling pathways in the oral cavity and the GI system that potentially influence food preference and acceptance. The information generated from the oral cavity is forwarded to the brain via the sensory afferent fibres [66,67,68]. Taste information is then transmitted to the primary taste cortex [69,70,71]. The neurons in the primary taste cortex relay information to pathways involved in the central processing of the food reward along the dopaminergic midbrain [69,70,71]. Neurons within the dopaminergic midbrain subsequently inform other brain regions that are involved in the food reward system. The brain regions known to date that are involved in the food reward system include the orbitofrontal cortex, caudate nucleus and amygdala [71]. The activation of the brain centres involved in the reward pathway consequently releases dopamine, a neurotransmitter frequently linked with reward [71]. Brain autonomic centres may also relay and receive information with the GI system via the vagal nerve to coordinate with satiety hormones to prepare the digestive system for the incoming carbohydrate-rich food [71,72]. Although hypothetical at this stage, the body’s food reward system, along with the sugar sensing mechanisms in the oral cavity and GIT, may play a crucial role in the regulation of eating behaviour, as well as possibly controlling the amount of dietary energy an individual consumes [73]. The question now is whether HIS can impact the central taste and reward pathways similarly to caloric sugars in the brain, given that HIS are able to activate TRC in the oral cavity, or whether the brain itself is able to identify HIS and, thus, sends information to the gut system not to activate SGLT-1. That is, does one feel the same level of satisfaction and pleasure upon ingesting artificially sweetened food as compared to sugar or does appetite increase or satiety decrease upon ingestion of foods containing HIS?

### 3.6. Are HIS Equally as Rewarding to the Brain as Caloric Sugar?

Three studies using functional magnetic resonance imaging (fMRI) data investigating if HIS are equally as rewarding to the brain as caloric sugar revealed some interesting findings. In a study by Smeets *et al.* [74], 20 healthy men were asked to rate the satisfaction and sweetness of solutions sweetened with either sucrose or a HIS while undergoing fMRI scans. It was found that the human brain responds differentially to sucrose and HIS, particularly in the activation of striatum (*i.e*., striatum, a brain region involved in the reward pathway, was not activated upon ingestion of an artificially sweetened solution in comparison to sucrose) [73]. Similarly, in another study by Frank *et al.* [75], researchers found that both caloric sugar and HIS were able to activate the primary taste pathway in the brain, but only caloric sugars were able to activate a significant response from brain regions involved in the brain’s reward pathway. These findings suggest that perhaps the brain’s reward pathway is adapted to favour caloric sweeteners in comparison to HIS [73]. However, an interesting question is whether there are differences between habitual consumers and non-habitual consumers of HIS. A recent study by Green and Murphy [76] found that diet soda drinkers demonstrated greater overall activation to sweet taste in several reward processing brain regions to both caloric sugar and HIS, compared to a non-diet soda drinker group. Within the diet soda drinkers, there was no difference in the brain’s response to both caloric sugar and HIS [74,76]. The lack of response to sweet taste among habitual consumers of beverages sweetened with HIS can potentially be explained as a result of the repeated experience of sweetness without energy. It is also important to note that these studies do not convey causality.

## 4. Possible Functions of Sweet Taste Perception

The latest progresses in our knowledge surrounding sweet taste detection mechanisms for caloric sugars and HIS within the mouth, the brain and the GIT [24,25,26,27,28,29,30,31,32,33,34,35,36,37,38,39,40,49,55,66,67,68,69,70,71,72,73], combined with their fundamental role in regulating appetite and, thus, dietary energy intake [49,55,56,61,65,71,72], suggest that abnormalities in any or several of these nutrient sensors may be an underlying factor of why some individuals consume more energy [77,78,79]. As such, variations in oral sweet taste detection (initial contact with food) towards caloric sugars and HIS and how these may influence appetite (satiation and satiety) are of high interest. Although generally hypothetical at the present time, the links between sweet taste function, satiation, satiety and weight suggest an intriguing model to understand being overweight and obesity.

### Individual Differences in Oral Sweet Taste Sensitivity

As with all taste qualities, there is large individual variation in the capability to perceive sweet taste (sweet taste sensitivity) [80,81,82]. These large individual variations in the ability to perceive sweet taste may perhaps explain why some individuals, in particular those who are less sensitive to sweetness, consume more sugar and/or have greater dietary intake in comparison to those who are more sensitive to sweetness [81]. Individuals who are less sensitive to sweetness are postulated to be at risk of long-term health outcomes, such as obesity, as they will need to consume more sugar to have the same taste sensation compared to those who are more sensitive [81]. For example, an individual who is very sensitive to sweetness may only need to add a teaspoon of sugar to their coffee in order to achieve the desired sweetness sensation. Conversely, a less sensitive individual may need to add more teaspoons of sugar in order to achieve a similar level of sweetness sensation, thus increasing total energy intake. Therefore, studying the individual variance in sweet taste sensitivity may be an important underlying factor to understand why some individuals may consume excess dietary sugar.

Variations in sweet taste sensitivity are likely to be influenced by environmental and genetic factors. A recent study investigating the associations between common variation in genes encoding the human sweet taste system and the individual variation in sweet taste sensitivity among 160 unrelated individuals found that the taste threshold and suprathreshold sensitivities of participants for sucrose were significantly associated with the genetic variation occurring in the GNAT3 gene (which encodes for α-gustducin) [83]. Interestingly, the genetic variations of the GNAT3 gene also explained 13% of the variation in sweet taste perception [83]. Another study of female monozygotic and dizygotic twins reported that genetics accounts partly for the ability to perceive the intensity of a sweet (sucrose) solution and for the liking of sweet foods [84], suggesting that liking of sweet-tasting foods is a multifactorial, polygenic trait [85].

Recent studies have also suggested that the individual differences in detecting or recognizing sweetness may perhaps be influenced by sensitivity to other taste qualities, such as bitter taste [86,87,88]. For example, Zhang *et al.* [86] found an inverse correlation in the relationship between the detection threshold for sucrose and the fungiform papillae density (*i.e*., a higher density of fungiform papillae is associated with individuals that are more sensitive to sucrose). Given that the most commonly used HIS, such as aspartame, acesulfame K, sucralose and rebaudioside A, have been reported to have a long-lasting bitter after-taste [89,90], it is essential to investigate further if consumption of HIS is driven by an individual’s sensitivity to both sweet and bitter taste.

In addition to an individual’s genetic predisposition, taste sensitivity may be influenced by environmental factors, such as dietary intake. For instance, taste detection thresholds for sucrose were found to be significantly lower after a 12-week, calorie-restricted weight loss program in obese females [91]. This suggests that weight loss may lead to an improvement in detecting sweet taste, which can be modified through changes in diet. Nevertheless, whether or not habitual diet can alter sweet taste sensitivity or *vice versa* is still unclear. In a recent study investigating the relationship between sweet intensity perception and dietary intake, no significant associations were found between perceived sweet intensity and dietary energy intake relating to sugar consumption [80]. However, in this study only a single measure of sweet taste was used, and perhaps, using another measure of sweet taste thresholds (*i.e*., detection threshold, recognition threshold and suprathreshold perception) could yield different outcomes [80].

## 5. Implications of Decreased Oral and GI Sweet Taste Sensitivity and Perception

### 5.1. Implications of Oral Sweet Taste Sensitivity on Body Mass Index

The evidence that disruption in the sweet taste threshold is associated with obesity is limited. Recent studies have suggested that individuals with a high body mass index (BMI) may not only taste sweet as being less intense than healthy weight individuals, but they may also have increased sweet preference [80]. Another study by Felstad *et al.* [92] has shown that people with a high BMI report lower pleasantness upon consuming sweet foods compared to people with a lower BMI. No differences in sweet taste detection threshold [93,94,95] or suprathreshold intensities [96,97] have been shown between different BMI groups. However, discrepancies between studies may be attributed to differences in the types of sugar used and/or psychophysical techniques used to measure sweet taste function [78].

### 5.2. Sweet Taste and Appetite

The influence of sweetness on appetite has been of interest over the past few decades. However, the specific role of sweet taste in appetite regulation is controversial (reviewed in [14]). For example, some studies, but not all, show that sweet taste in itself can stimulate hunger [98,99,100,101], whereas some failed to show an effect of sweet taste on appetite and food intake in subsequent meals [102,103,104,105,106]. It is possible that some studies show no effect of sweet taste on appetite, as there may be individual differences in sweet taste thresholds [101]. In a study by Tordoff and Alleva [101], the levels of sweetness of chewing gums were manipulated by using different concentrations of aspartame (HIS). Interestingly, it was found that the most effective sweetness level to increase hunger differed among most individuals [101]. In two other studies investigating the palatability of yogurts with different concentrations of aspartame (HIS) and sucrose, yogurt intake was found to be greater at the sweetness level most preferred by the individual [107,108]. Using a 24-hour food diary, subsequent food intake was also reported to be significantly greater following consumption of the yogurt with the preferred sweetness level [107,108].

### 5.3. Links between Oral Sweet Taste Perception of Caloric Sweeteners and High Intensity Sweeteners and Satiation

The process of satiation or fullness is, to a large degree, mediated by sensory processes, generated from the flavour attributes of foods (sight, taste and smell) [109]. When a food is eaten to satiety, the pleasantness of the flavour properties of that food decreases more than for other foods [110]. This is termed “sensory-specific satiety” [110]. Development of sensory-specific satiety is predominantly associated with sensory stimulus accompanying the ingestion of food, as opposed to the post-absorptive effects of consuming these foods [110]. A key factor that contributes to the cessation of the meal is thought to be the diminished pleasure from exposure to the flavour [14].

The influence of sweet protein flavours on satiation has sparked some interest over the past few decades. However, how different caloric sweeteners and HIS influence satiation is not fully clarified. A study by Rolls *et al.* [111] investigated the effects of sucrose and aspartame-sweetened (HIS) gelatines on appetite ratings and subsequent food intake using a cross-over design. Participants were instructed to consume either a high-calorie gelatine (sweetened with sucrose) or a lower-calorie version (sweetened with aspartame) [111]. Interestingly, there were no significant differences found between rated sensory-specific satiety, hunger, fullness and desire to eat following consumption of both versions of the gelatines [111]. Similarly, another study by Rolls *et al.* [103] further investigated if there were differences in terms of short-term appetite and food intake following consumption of drinks sweetened with either sucrose or aspartame (HIS) in men. The results suggest that there were no differences between consuming a sugar-sweetened beverage or an aspartame-sweetened (HIS) beverage in short-term hunger ratings and subsequent food intake [103]. A possible explanation of these two studies is that the oral sweet TRC were able to sense both sugar and aspartame (HIS), resulting in no subsequent effect on short-term hunger and appetite following consumption of sugar or aspartame-sweetened (HIS) drinks and gelatines.

Two other studies examining the effects of sweet taste on short-term hunger were conducted among habitual high and low consumers of beverages sweetened with HIS [112,113]. It was found that the effects of the sweet and non-sweet lunches on short-term hunger differed significantly in terms of individuals’ habitual consumption of sweet low-calorie drinks (*i.e*., an increase in short-term appetite ratings in response to sweet taste was demonstrated among low consumers of beverages sweetened with HIS, whereas high consumers did not show this increase) [112,113]. Consistent with what was previously mentioned, it is possible that the appetite ratings differed between habitual consumers and non-consumers of HIS drinks because of other contributing factors, such as adaptation (*i.e*., impaired sweet taste perception and sweet reward pathways following repetitive consumption of a particular sweet stimulus), which may influence food behaviours.

### 5.4. Links between Attenuated Oral Sweet Taste Perception, the Gastrointestinal System and Satiety

As previously mentioned, peripheral mechanisms for identifying sugars and HIS are located throughout the GIT, which may possibly influence appetite. Recent studies investigating the link between hormonal response in the GI system and being overweight and obesity have produced interesting findings. A study by Perry and Wang [114] suggested that these satiety hormonal responses (*i.e*., PYY, GLP-1 and GIP) are impaired in obese individuals, further raising the likelihood that perhaps energy intake may be poorly regulated among obese individuals due to the impairment in the GI appetite response [113]. Similarly, there is some evidence to suggest that differences in GI hormone secretion exist between lean and obese individuals [21]. For example, obese individuals reported a lower level of postprandial PYY and GLP-1 secretion compared to lean individuals, with levels returning to normal following weight loss through gastric bypass surgery [115,116,117]. Additionally, there is a possibility that obese individuals may experience an impaired oral sweet taste sensitivity in comparison to lean individuals [78], suggesting a coordinated alimentary canal response to sweet taste detection from the oral cavity to the GIT [31].

## 6. Conclusions

Increased energy intake, in particular, increased intake of sweet, energy-dense food, is thought to be one of the contributors to the rising rates in overweight individuals and obesity globally. Based on this idea, one popular way of combating the current obesity crisis is to reduce energy intake through HIS. Replacing sugar with HIS in food and beverages decouples sweetness from the energy value while maintaining sweetness. However, the role of sweet taste in energy intake and appetite regulation is controversial. The first part of this review focused on discussing the possible associations between sweet taste mechanisms within the oral cavity, GIT and the brain system towards both sugar and HIS. The identification of sweet TRC in both the oral cavity and GIT produced important insights into the mechanisms underlying sweet taste perception for both sugar and HIS. Collectively, the literature investigating the sensing mechanisms of sweet taste detection in both the oral cavity and GIT suggest that perhaps SGLT-1 is more responsible in the gut compared to the oral cavity, where sweet TRC are the primary sugar/HIS sensors. Based on *in vitro* studies, HIS failed to activate SGLT-1, and thus, no secretion of satiety hormones was observed upon ingestion of HIS in the GIT. It is important to note, however, that this mechanism may not extend to humans, since the studies were based on animal GI cells. Therefore, the full description of SGLT-1 in human studies is of relevance due to their apparent involvement in the GI functions and appetite regulation. The available data from brain studies comparing HIS and sugars have also revealed interesting findings regarding habitual consumers and non-habitual consumers of HIS. It was found that compared to a non-habitual consumer of HIS, a habitual consumer of HIS had greater overall activation in the brain reward pathways to both sugar and HIS. The reward pathways of a non-habitual consumer of HIS are generally not activated when consuming HIS as opposed to sugar, suggesting that the HIS may impair and adapt the brain’s capability to detect or sense nutrients. However, it is uncertain at this stage why the brain will adapt to sense HIS as nutrients. It would be interesting if future studies investigate whether obese habitual users of HIS had impairments in their oral, brain and gut sensing mechanisms of sweetness. This would then provide strong evidence linking these mechanisms into a unified theory of obesity development.

It has been suggested in several papers [77,78,79] that perhaps abnormalities in any or several of these nutrient-sensing mechanisms (*i.e*., sweet taste detection mechanism in the oral cavity, the brain and GIT) may be important to understand why some individuals consume more calories. Hence, understanding individual differences in detecting and recognizing sweetness towards caloric sugars and HIS and how these mechanisms may influence appetite (sensory-specific satiety and satiety) is important and needs to be verified in the future. Although largely hypothetical at this stage, the review of the literature investigating the potential links between sweet taste function, satiation, satiety and BMI reveals an interesting model and potential factor to understand being overweight and obesity.
